# Breast Cancer in Primary Healthcare: Clinical and Epidemiological Characteristics Associated With Age at Diagnosis and Metastatic Status

**DOI:** 10.7759/cureus.115378

**Published:** 2026-08-28

**Authors:** Senada Selmanovic, Amila Vujadinovic, Ezaneta Merdanovic, Branislav Milic, Danijel Gajic, Emina Bajric Custo, Sabina Ademovic Dzambic

**Affiliations:** 1 Family Medicine, Health Center "Dr. Mustafa Šehović", Tuzla, BIH; 2 Family Medicine, Health Center Gračanica, Gračanica, BIH; 3 Psychiatry, Health Center Orašje, Orašje, BIH; 4 Family Medicine, Health Center Orašje, Orašje, BIH; 5 Family Medicine, Health Center Lukavac, Lukavac, BIH

**Keywords:** breast cancer, breastfeeding, menopause, obesity, risk factors

## Abstract

Background: This study aimed to explore the demographic, reproductive, lifestyle, screening, and clinical characteristics of breast cancer patients and assess their associations with age at diagnosis and metastatic status at diagnosis in a primary healthcare setting.

Methods: A retrospective exploratory study was conducted using medical records of all eligible female breast cancer patients (N = 50) registered across five family medicine teams in Tuzla and diagnosed up to August 2025. As the study included the complete eligible population within the predefined primary healthcare setting, no a priori sample size calculation was performed. Data were analyzed using descriptive statistics, independent-samples t-tests with Welch’s correction when appropriate, one-way analysis of variance (ANOVA), Pearson’s chi-square test, and exact tests for sparse contingency tables. Statistical significance was defined as p < 0.05.

Results: The mean age at diagnosis was 58.90 ± 12.19 years. Tumor mass was most frequently detected via self-examination (n = 20, 40.0%) and mammography screening (n = 17, 34.0%). Mean age at diagnosis differed significantly across detection modes (one-way ANOVA, F = 4.712, p = 0.003). Descriptively, the mean age at diagnosis was 56.25 ± 11.01 years among patients whose tumors were detected by self-examination and 63.53 ± 11.65 years among those detected by mammography. Similarly, patients without prior mammography (n = 28, 56.0%) were diagnosed nearly a decade earlier than those who underwent prior screening (n = 22, 44.0%) (54.61 ± 13.75 vs. 64.36 ± 6.93 years; Welch's t = -3.221, df = 40.23, p = 0.002). Patients with lymphatic metastases were diagnosed at a significantly younger age than those without metastases (51.17 ± 7.52 vs. 61.56 ± 12.71 years; p = 0.011). Breastfeeding history was documented in seven patients (14.0%), while breastfeeding information was unavailable for the remaining patients. No statistically significant association was observed between late menopause and metastatic status at diagnosis (p = 0.053). Regarding modifiable lifestyle characteristics, overweight (n = 18, 42.9%) and obesity (n = 14, 33.3%) were present in 76.2% of evaluated patients (n = 42), while active smoking was recorded in 22.0% (n = 11) participants.

Conclusion: In this exploratory primary healthcare cohort, age at diagnosis differed across modes of detection, while patients with lymphatic metastases were younger at diagnosis than those without metastases. No statistically significant association was observed between late menopause and metastatic status. Overweight and obesity were common among patients with available BMI data, while breastfeeding history was insufficiently documented for reliable evaluation. These findings should be interpreted cautiously given the small sample size and retrospective design and may help inform larger studies on breast cancer presentation, screening, and early detection.

## Introduction

According to World Health Organization (WHO) data, breast cancer is the leading malignancy among women worldwide. It predominantly affects older women, with marked global disparities in mortality rates. Although highly penetrant gene mutations (BRCA1/2) and a family history significantly increase the risk, the majority of affected women have no known genetic predisposition. Consequently, female sex and older age (over 40 years) remain the most dominant risk factors, along with a significant influence of modifiable lifestyle habits and reproductive history [1].

Female sex is one of the primary factors associated with an increased risk of breast cancer, mainly due to enhanced hormonal stimulation. Unlike men, who exhibit negligible estrogen levels, women possess breast tissue cells that are highly sensitive to hormones, particularly estrogen and progesterone, and to any disruptions in their balance. Circulating estrogens and androgens are positively correlated with an increased risk of breast cancer [2]. Currently, approximately 80% of breast cancer patients are over the age of 50, while more than 40% are older than 65 years [3]. The risk of developing breast cancer increases by 1.5% at age 40, 3% at age 50, and more than 4% at age 70 [4].

Family history represents a significant non-modifiable risk factor, with first-degree relatives affected by breast cancer doubling the risk of developing the disease. This risk increases further with a higher number of affected family members, as well as with a younger age at diagnosis of both the relative and the patient [5]. According to epidemiological data, obesity is associated with a higher probability of developing breast cancer. This is most pronounced in obese postmenopausal women who tend to develop estrogen receptor-positive breast cancer [6].

Carcinogens from tobacco smoke reach the breast tissue, where they increase the risk of mutations in oncogenes and tumor suppressor genes (particularly the p53 gene). Accordingly, both active and passive smoking significantly contribute to the initiation of pro-carcinogenic processes. Additionally, a longer smoking history, as well as smoking prior to the first full-term pregnancy, represent major risk factors that are particularly pronounced in women with a positive family history of breast cancer [7]. Breastfeeding is associated with a protective effect against breast cancer in multiparous women. Every 12 months of lactation can reduce the relative risk of developing this disease by 4.3%, while cumulative breastfeeding for a year or longer can reduce the risk by up to 28%. It is estimated that women who have breastfed at any point in life have a 14% lower probability of developing the disease compared to those who have never breastfed, with current global breastfeeding rates expected to prevent approximately 20,000 breast cancer deaths annually [8].

Mammography represents the gold standard in the early detection of breast cancer, directly contributing to a reduction in mortality rates. In addition to mammography, breast self-examination, clinical breast examination (CBE), and ultrasound diagnostics are also utilized in clinical practice. High accuracy of diagnostic methods is crucial, given that false-positive findings can lead to psychological distress and exposure to invasive procedures, whereas false-negative findings delay diagnosis and potentially worsen prognosis. Although the role of mammography in reducing mortality is indisputable, clinical studies point to variations in its sensitivity and efficiency depending on patient age (e.g., due to dense breast parenchyma in younger women), highlighting the need for further refinement of screening protocols [9]. Metastatic progression presents one of the greatest therapeutic challenges in breast cancer management. Cases of distant disease spread most frequently involve the lungs, liver, bones, and brain, while specific biomarkers for organ-specific metastasis remain the subject of intensive research [10]. In the local healthcare setting, family medicine teams provide longitudinal care and maintain records on screening history, lifestyle and reproductive characteristics, and relevant clinical information. Primary care therefore offers a practical real-world setting for examining patterns of breast cancer detection and clinical presentation within the local population.

The primary objective of this study was to explore the demographic, anthropometric, reproductive, lifestyle, and clinical characteristics of breast cancer patients in a primary healthcare setting and to assess their associations with metastatic presentation and age at diagnosis.

The secondary objectives were to examine differences in age at diagnosis according to the mode of breast cancer detection; to describe the distribution of selected demographic, reproductive, and lifestyle characteristics, including BMI, family history, smoking status, breastfeeding history, age at menarche, and age at menopause; and to explore their associations with metastatic presentation.

## Materials and methods

Study design

A retrospective exploratory study was conducted in August 2025 across five family medicine teams at the Public Health Institution Health Center “Dr. Mustafa Šehović” in Tuzla. The study included all eligible female patients with a confirmed diagnosis of breast cancer who were actively registered within the selected family medicine teams and whose breast cancer diagnosis had been documented up to August 2025. Data were collected by reviewing the Registries of Patients with Malignant Diseases and the available medical records (patient charts).

The retrospective exploratory design was selected because the study aimed to characterize routinely documented demographic, reproductive, lifestyle, screening, and clinical features among patients already diagnosed with breast cancer and to explore their association with age at diagnosis and metastatic status at diagnosis using existing medical records.

All eligible breast cancer patients registered within the five predefined family medicine teams and diagnosed up to August 2025 were included. Therefore, no a priori sample size calculation was performed, as the study did not draw a sample from a larger accessible cohort but included the complete available eligible population within the selected primary healthcare setting. The final sample of 50 patients thus represents complete enumeration of this defined population. Given the limited sample size, all inferential analyses were considered exploratory and hypothesis-generating.

Subjects and data collection

The study sample consisted of 50 female patients with a confirmed diagnosis of breast cancer. The specific inclusion and exclusion criteria applied during sample selection are summarized in Table 1.

**Table 1:** Inclusion and exclusion criteria for study participant selection

Inclusion criteria	Exclusion criteria
Confirmed histopathological diagnosis of breast cancer	Male sex with breast cancer diagnosis
Female sex	Non-registration with the selected family medicine teams
Active registration within one of the five selected family medicine teams	Patients with another primary carcinoma

Due to the retrospective nature of data collection from medical records, certain anamnestic variables (such as age at menarche) were not fully documented for all patients; therefore, relevant analyses of specific reproductive parameters were conducted on sub-samples with available data. Demographic parameters (current age and age at diagnosis) and clinical characteristics, including the mode of detection (screening vs. symptomatic presentation), presence of metastases, and coverage by preventive examinations, were extracted from the medical records. Selected demographic, reproductive, lifestyle, screening, and clinical characteristics were analyzed, including family history of breast cancer, age at menarche and menopause, breastfeeding history, BMI, smoking status, prior mammography, mode of detection, age at diagnosis, and metastatic status at diagnosis.

Demographic, reproductive, lifestyle, screening, and clinical variables were extracted from the available medical records. Prior mammography was defined as any documented mammographic examination performed before the diagnosis of breast cancer.

Mode of detection was defined as the method documented in the medical record as leading to the initial detection of the breast lesion and was categorized as self-examination, breast ultrasound, mammography, chest computed tomography (CT), or not recorded. Smoking status was categorized as never smoker, current smoker, or former smoker according to the smoking history documented in the medical record. A current smoker was defined as a patient documented as actively smoking at the time covered by the medical record, a former smoker as a patient with a documented previous history of smoking who had subsequently stopped, and a never smoker as a patient with no documented history of active smoking.

Positive family history was defined as a documented history of breast cancer among blood relatives, whereas absence of such a history was classified as negative family history. Early menarche was defined as the onset of menstruation before 12 years of age. Late menopause was defined as menopause occurring after 55 years of age.

Metastatic status was defined according to the presence or absence of metastases documented at the time of breast cancer diagnosis. Patients were categorized as having no metastases, lymphatic system metastases, or lymphatic system metastases accompanied by metastases to other organs. Breastfeeding history was classified as documented breastfeeding when explicitly recorded in the medical record. In the absence of reliable documentation regarding breastfeeding history, the information was classified as unknown/missing rather than interpreted as confirmed absence of breastfeeding. For variables with incomplete documentation, analyses were performed using available cases only, and the corresponding denominator was reported where applicable.

Statistical analysis

Data were analyzed using IBM SPSS Statistics for Windows, Version 26.0 (Released 2018; IBM Corp., Armonk, NY, USA). Continuous variables were assessed for normality using the Kolmogorov-Smirnov and Shapiro-Wilk tests, together with examination of skewness and kurtosis. Continuous variables are presented as mean and standard deviation (SD), as well as median, interquartile range (Q1-Q3), and minimum-maximum values where appropriate. Categorical variables are presented as absolute frequencies (N) and percentages.

Differences in mean values of continuous variables between two independent groups were assessed using the independent-samples t-test. Homogeneity of variances was evaluated using Levene’s test, and Welch’s correction was applied when the assumption of equal variances was violated. One-way analysis of variance (ANOVA) was used to compare mean age at diagnosis across more than two independent groups.

Associations between categorical variables were assessed using Pearson’s chi-square test when its assumptions were satisfied. Expected cell frequencies were examined for contingency-table analyses. Fisher’s exact test, or the appropriate exact extension for larger contingency tables, was used when expected cell frequencies were too small to satisfy the assumptions of Pearson’s chi-square test. Cramer’s V was reported as a measure of effect size where applicable.

Linear-by-linear association testing was not used in the revised analyses because the relevant variables did not meet the required ordinal structure for both variables.

All statistical tests were two-sided, and a p-value < 0.05 was considered statistically significant. Findings with p ≥ 0.05 were considered statistically non-significant.

Missing or undocumented information was treated as missing data rather than being interpreted as the absence of the respective characteristic. Analyses involving variables with incomplete documentation were performed using available cases only, and denominators were reported accordingly.

Ethical considerations

The study was conducted in full compliance with the principles of medical ethics, the Code of Deontology, and the Declaration of Helsinki. Official approval for conducting the study was granted by the Ethics Committee of the Public Health Institution Health Center "Dr. Mustafa Šehović" Tuzla. Participation in the study was voluntary; all patients whose data were used were informed beforehand about the study objectives and personally signed the "Informed Consent Form for Participation in Medical and Scientific Research." Privacy and data confidentiality were strictly guaranteed through complete sample anonymization.

## Results

Descriptive statistical analysis of age and BMI is presented in Table 2. Analysis of patient age at the time of the study indicates a predominant representation of older patients (69.62 ± 11.59 years). At the time of diagnosis, the mean age was 58.90 ± 12.19 years, with half of the patients diagnosed between the ages of 50 and 68. Measures of skewness and kurtosis indicate that the distribution of age at diagnosis does not deviate significantly from a normal distribution.

**Table 2:** Baseline demographic, anthropometric, and anamnestic characteristics of the study participants

	Age at the time of study	Age at diagnosis	BMI (kg/m²)
N	50	50	42
Mean	69.62	58.90	27.36
Median (50%)	71.50	59.00	27.25
Standard deviation	11.586	12.189	3.920
Minimum/maximum	44/94	35/91	20/36
Range	50	56	17
Quartiles (25%/75%)	63.50/77.25	50.00/68.00	24.75/30.00
Skewness	-0.336	0.192	-0.041
Kurtosis	-0.096	-0.147	-0.507

Assessment of nutritional status (available for 42 patients) reveals a high prevalence of elevated body weight. The mean BMI was 27.36 ± 3.92, showing a symmetrical data distribution. Just under a quarter of the participants (N = 10; 23.8%) had a normal body weight, whereas the majority fell into the categories of overweight (N = 18; 42.9%) or obese (N = 14; 33.3%) (Figure 1A).

**Figure 1:**
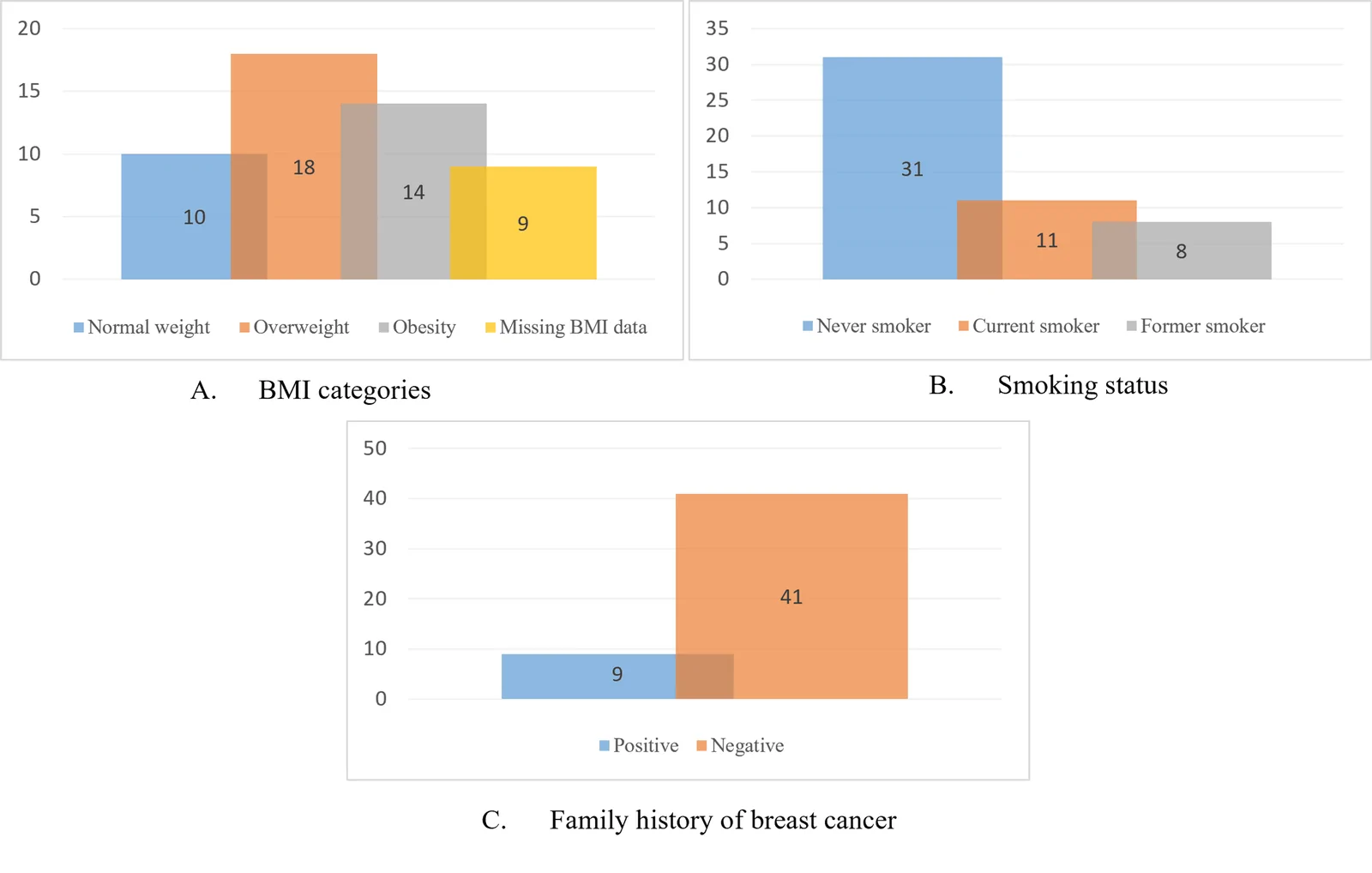
Distribution of selected participant characteristics: (A) body mass index categories, (B) smoking status, and (C) family history of breast cancer

Analysis of the participants' smoking status (Figure 1B) indicates that the largest proportion of the sample consisted of non-smokers (62%, N = 31), while 22% (N = 11) of the participants regularly smoked. A notable proportion of participants (16%, N = 8) were former smokers, reflecting changes in smoking habits within the studied population.

Analysis of the participants' family history indicates that 82% (N = 41) of the participants had a negative family history of breast cancer, whereas a positive family history was recorded in 18% (N = 9) of the participants (Figure 1C).

Data on breastfeeding extracted from medical records are presented in Table 3, showing that the majority of participants (N = 43, 86%) had no recorded history of breastfeeding, while 7 (14%) had breastfed.

**Table 3:** Frequency distribution of breastfeeding among study participants

Breastfeeding history	Frequency (N)	Percentage (%)
Unknown/not documented	43	86.0
Documented breastfeeding	7	14.0
Total	50	100.0

A descriptive summary of menstrual cycle parameters and menopause is presented in Table 4. Data regarding age at menarche were available for 45 patients, with a mean age of 13.51 ± 1.41 years. Early menarche (before age 12), a known risk factor due to prolonged estrogen exposure, was recorded in five (11.1%) of the participants. Age at menopause onset was analyzed in 40 postmenopausal patients, with a mean of 49.25 ± 5.37 years. Late menopause (after age 55), which also represents a risk factor due to an extended period of hormonal exposure, was observed in 12.5% of the women (N = 5).

**Table 4:** Descriptive statistics for age at menarche and menopause

Parameter	Menarche	Menopause
Number of participants (N)	45	40
Missing data	5	10
Mean (years)	13.51	49.25
Median (50%)	14.00	50.00
Standard deviation	1.408	5.372
Minimum/maximum	11/16	36/60
Range	5	24
Quartiles (25%/75%)	12.50/15.00	45.00/53.00
Skewness	-0.279	-0.359
Kurtosis	-0.816	-0.287

No statistically significant association was observed between late menopause and metastatic status at diagnosis (p = 0.053) (Table 5).

**Table 5:** Correlation between late menopause and metastases in women with breast cancer

Late menopause	No metastases	Lymphatic system	Lymphatic + other organs	Total	p-value
No	26	7	2	35	0.053	
Yes	2	1	2	5		
Total	28	8	4	40		

Detection mode analysis showed self-examination in 20 (40.0%), mammography in 17 (34.0%), breast ultrasound in 10 (20.0%), chest CT in 1 (2.0%), and an unrecorded detection method in 2 (4.0%). Mean age at diagnosis differed significantly across detection modes (one-way ANOVA, F = 4.712, p = 0.003) (Table 6).

**Table 6:** Descriptive statistics of age at diagnosis according to the method of breast cancer detection *Statistical significance: p ≤ 0.05.

Detection method	N	Mean (age)	Standard deviation	Minimum	Maximum	p-value
Self-examination	20	56.25	11.01	38	78	0.003*
Breast ultrasound	10	58.70	8.65	41	74	
Mammography	17	63.53	11.65	44	91	
Chest CT	1	82.00	-	82	82	
Not recorded	2	35.50	0.71	35	36	
Total	50	58.90	12.19	35	91	

Patients without prior mammography (n = 28, 56.0%) were diagnosed significantly earlier than those who underwent prior screening (n = 22, 44.0%) (54.61 ± 13.75 vs. 64.36 ± 6.93 years; Welch's t = -3.221, df = 40.23, p = 0.002) (Table 7).

**Table 7:** Association between preventive mammography screening and mean age at diagnosis *Statistical significance: p ≤ 0.05.

Mammography prior to diagnosis	N	Mean age at diagnosis	Standard deviation	p-value
No	28	54.61	13.75	0.002*
Yes	22	64.36	6.93	

Most participants (68%, N = 34 ) had no recorded metastases, whereas 24% (N = 12) presented with lymphatic metastases, and 8% (N = 4) had metastases in both lymph nodes and other organs. Patients without metastases were diagnosed at a significantly older mean age (M = 61.56, SD = 12.71) than those with lymphatic involvement (M = 51.17, SD = 7.52; t = 3.379, df = 33.14, p = 0.011). Patients with lymphatic metastases were diagnosed at a significantly younger mean age than patients without metastases, with a mean difference of 10.39 years (Table 8).

**Table 8:** Presence of metastases and mean age of participants *Statistical significance: p ≤ 0.05.

Metastases	N	Mean age at diagnosis	Standard deviation	p-value
No metastases	34	61.56	12.71	0.011*
Lymphatic system metastases	12	51.17	7.52	

## Discussion

In this study, we evaluated demographic data, lifestyle habits, gynecological history, and clinical characteristics of the study participants. The mean age of the patients in our study was 69.62 years. The observed age structure indicates a marked predominance of the older population within our sample.

Comparisons with published literature indicate differences in age at diagnosis across study populations. Khalaf et al. reported a mean age of 48 years, whereas the mean age at diagnosis in our cohort was higher. This difference may reflect variations in population demographics, life expectancy, healthcare utilization, and methodological characteristics of patient identification and registry-based data collection in primary care settings [11].

However, the clinical expression of the disease varies significantly across age groups. Our finding that patients with confirmed lymph node metastases were, on average, 10 years younger than those without nodal involvement is consistent with reports suggesting that breast cancer in younger women may present with less favorable clinical characteristics. Because younger women frequently present with higher mammographic breast density and routine mammography is not recommended at a young age, it can be hypothesized that this contributes to the more frequent detection of tumors via self-examination in younger participants. As emphasized by recent global analyses (such as those based on GLOBOCAN 2022 data), disparities in survival among younger patients are particularly pronounced in developing and transitioning countries, where organized screening programs tailored to younger populations and early coverage with diagnostic ultrasound are lacking. These observations support continued awareness of breast changes and timely clinical evaluation, alongside age-appropriate screening [12].

Family history represents one of the most important non-modifiable risk factors. In our study, 18% (N = 9) of patients had a verified positive family history of breast cancer, reflecting a documented familial burden of breast cancer within a subset of the cohort [13].

Regarding modifiable lifestyle characteristics, contemporary literature places increasing emphasis on nutritional status and obesity. Previous studies have shown that postmenopausal women with higher BMI may have an increased risk of developing breast cancer and may present with less favorable tumor characteristics, including larger primary lesions and more frequent regional lymph node involvement. In our cohort, overweight and obesity were common, being present in 76.2% of patients with available BMI data. However, because the present study included only patients with breast cancer and lacked a non-cancer comparison group, these descriptive findings cannot establish obesity as a risk factor for breast cancer development in this population [14].

In our study, 62% of patients were never smokers, 22% were current smokers, and 16% were former smokers. Although smoking has traditionally not been considered a primary cause of breast cancer, observational epidemiological data point to a potential link with carcinogenesis in breast tissue. Comprehensive meta-analyses involving up to 90 studies have suggested that cigarette smoking is associated with an increased risk of developing breast cancer by 7% to 8% compared with non-smokers. These descriptive findings support the broader public health relevance of smoking cessation, although the present study was not designed to assess smoking as a causal risk factor for breast cancer [15].

Beyond primary prevention and early detection, lifestyle interventions are also relevant to cancer survivorship and quality of life. Recent systematic reviews and meta-analyses have demonstrated beneficial effects of physical activity on health-related quality of life among cancer patients and survivors, including specifically among patients and survivors of breast cancer. These findings support the broader integration of structured physical activity, weight management, and lifestyle counseling into survivorship care, while recognizing that the present study was not designed to evaluate the effects of such interventions [16].

Breast self-examination and a healthy lifestyle form essential components of breast cancer prevention and early detection. A meta-analysis of 11 randomized trials demonstrated that women aged 50-70 experienced a significant reduction in breast cancer mortality following mammographic screening. However, the reduction in mortality was not statistically significant in women aged 40-49. These results support the key role of organized mammographic screening programs in reducing mortality. Concurrently, continuous education on self-examination and fostering awareness of one’s own breast tissue anatomy may enable women to recognize new nodular or structural changes more easily, directly encouraging earlier presentation to a physician [17]. Regional lymph node involvement, particularly axillary, is generally associated with a poorer prognosis and a lower likelihood of complete cure via surgical intervention alone, as it often reflects disease spread beyond the primary site [18].

Breastfeeding history was documented in only 14% (N = 7) of patients, whereas information was unavailable or not documented in the remaining 86% (N = 43). Therefore, the prevalence of breastfeeding and its association with metastatic status could not be reliably assessed in this cohort. Previous studies have reported a protective association between breastfeeding and breast cancer risk. For example, Navarro-Ibarra et al. (N = 162) reported an adjusted OR of 0.34 (95% CI: 0.12-0.92) [19]. However, direct comparison with our cohort is limited by the substantial amount of missing breastfeeding data. The broader literature nevertheless supports breastfeeding as a beneficial reproductive and public health practice [8,19].

Analysis of reproductive parameters in our sample provides insight into total lifetime exposure to endogenous hormones. A large global meta-analysis confirmed that earlier age at menarche is associated with an increased risk of breast cancer. No statistically significant association was observed between age at menarche and age at diagnosis in this cohort. Conversely, age at menopause in our sample closely mirrored global averages. Late menopause is well-documented in the literature as increasing disease risk, particularly for hormone-receptor-positive and lobular tumors, due to prolonged hormonal stimulation. In our study, late menopause was not significantly associated with metastatic status at diagnosis. Given the small subgroup size, this finding should be interpreted cautiously [20].

The main strength of this study lies in its primary healthcare setting, providing real-world descriptive and clinical data derived from active family medicine registries. The inclusion of all eligible breast cancer patients registered within the selected family medicine teams also reduced the potential for selective inclusion within the defined study population.

Several limitations should, however, be considered when interpreting the findings. First, the relatively small sample size (N = 50) limited the statistical power of the analyses, particularly for small subgroups such as women with late menopause and those with documented breastfeeding history. Sparse cell counts in several contingency-table analyses may also reduce the reliability of some statistical estimates. In addition, multiple statistical comparisons were performed within a limited sample, increasing the possibility of chance findings.

Second, the absence of a non-cancer control group prevents estimation of breast cancer risk or protective effects of the examined characteristics. Third, the retrospective collection of data from medical records resulted in incomplete documentation for several variables, including breastfeeding history and BMI. Historical information may also have been affected by recall bias or incomplete recording.

Furthermore, potentially important confounding factors, including tumor subtype, disease stage, histological characteristics, parity, detailed reproductive history, genetic factors, and treatment-related variables, were not controlled for in the present analyses. Therefore, the observed associations should be interpreted cautiously and considered exploratory and hypothesis-generating rather than confirmatory. Despite these limitations, the study provides preliminary local data that may help inform future research in larger and more comprehensively characterized populations.

## Conclusions

This exploratory study identified several characteristics associated with the clinical presentation of breast cancer within the analyzed primary healthcare population. Patients with lymphatic metastases at diagnosis were significantly younger than those without metastases, while no statistically significant association was observed between late menopause and metastatic status. Due to incomplete documentation of breastfeeding history, its association with metastatic status could not be reliably evaluated. The high reliance on self-examination and mammography for initial tumor detection further highlights the importance of continued screening and public health awareness regarding early diagnosis.

Overweight and obesity were common among patients with available BMI data; however, these descriptive findings should not be interpreted as evidence of causality or breast cancer risk. The results may provide useful local clinical context and support continued attention to screening, early detection, and healthy lifestyle counseling. Given the retrospective design, small sample size, and absence of a non-cancer comparison group, the findings should be considered exploratory and hypothesis-generating and require confirmation in larger studies.
